# Multilevel Multiobjective Particle Swarm Optimization Guided Superpixel Algorithm for Histopathology Image Detection and Segmentation

**DOI:** 10.3390/jimaging9040078

**Published:** 2023-03-29

**Authors:** Anusree Kanadath, J. Angel Arul Jothi, Siddhaling Urolagin

**Affiliations:** Department of Computer Science, Birla Institute of Technology and Science Pilani, Dubai International Academic City, Dubai P.O. Box 345055, United Arab Emirates; p20180904@dubai.bits-pilani.ac.in (A.K.); siddhaling@dubai.bits-pilani.ac.in (S.U.)

**Keywords:** nature-inspired algorithms, particle swarm optimization, multiobjective algorithms, image segmentation, thresholding, histopathology

## Abstract

Histopathology image analysis is considered as a gold standard for the early diagnosis of serious diseases such as cancer. The advancements in the field of computer-aided diagnosis (CAD) have led to the development of several algorithms for accurately segmenting histopathology images. However, the application of swarm intelligence for segmenting histopathology images is less explored. In this study, we introduce a Multilevel Multiobjective Particle Swarm Optimization guided Superpixel algorithm (MMPSO-S) for the effective detection and segmentation of various regions of interest (ROIs) from Hematoxylin and Eosin (H&E)-stained histopathology images. Several experiments are conducted on four different datasets such as TNBC, MoNuSeg, MoNuSAC, and LD to ascertain the performance of the proposed algorithm. For the TNBC dataset, the algorithm achieves a Jaccard coefficient of 0.49, a Dice coefficient of 0.65, and an F-measure of 0.65. For the MoNuSeg dataset, the algorithm achieves a Jaccard coefficient of 0.56, a Dice coefficient of 0.72, and an F-measure of 0.72. Finally, for the LD dataset, the algorithm achieves a precision of 0.96, a recall of 0.99, and an F-measure of 0.98. The comparative results demonstrate the superiority of the proposed method over the simple Particle Swarm Optimization (PSO) algorithm, its variants (Darwinian particle swarm optimization (DPSO), fractional order Darwinian particle swarm optimization (FODPSO)), Multiobjective Evolutionary Algorithm based on Decomposition (MOEA/D), non-dominated sorting genetic algorithm 2 (NSGA2), and other state-of-the-art traditional image processing methods.

## 1. Introduction

Histopathology is a branch of biology which deals with the examination of diseased tissues under a microscope to diagnose diseases [1]. Histopathology is useful in diagnosing cancerous conditions, identifying the stage of cancer and other inflammatory diseases. Though histopathology image analysis by pathologists plays a critical role in the early diagnosis of cancer, analysing a huge amount of tissue images under a microscope is a tedious and time-consuming task. This could further be hindered due to ambiguous regions in the histopathology images, inaccuracies in the devices, and human error. In recent times, digital pathology coupled with advancements in computer-aided diagnosis (CAD) systems is revolutionizing the area of histopathology. CAD systems are automated image analysis systems that can assist medical practitioners. Detection and segmentation of regions of interest (ROIs) from whole-slide images (WSIs) are some of the core operations of CAD systems in histopathology image analysis.

The literature contains a variety of histopathology image segmentation techniques, including traditional methods as well as deep learning methods used in CAD systems [2]. Traditional image processing methods, such as thresholding, region growing, clustering, watershed, active contour models, neural networks, and wavelet transforms, have been widely used for histopathology image segmentation [3,4,5]. Recently, deep learning algorithms have exhibited their capacity to capture essential features for efficient image segmentation; however, the performance of deep learning models is heavily dependent on the quality and quantity of training data and the amount of training time. The lack of huge annotated histopathology image data is a major challenge in applying deep learning models for histopathology image segmentation [4].

Thresholding is a simple and effective traditional image segmentation technique. In thresholding, an input image is divided into multiple images containing various regions based on threshold values. In multilevel thresholding, *k* threshold values are used to divide the image into k+1 images with several distinct regions. The optimal threshold is the best intensity value that segments the ROIs from the image accurately. Traditionally, the optimal threshold is identified by applying each intensity value of the image as a threshold value and then comparing the segmentation result. Thus, identifying the optimal threshold value is a complex and time-intensive task.

Another effective method widely used in the literature is to treat the problem of finding the optimal thresholds as an optimization problem and solve it using nature-inspired optimization algorithms. If the optimization problem uses single objective function it is called as a single-objective optimization problem (SOP). An optimization problem with more than one objective function is called a multiobjective optimization problem (MOP) [6,7,8]. The particle swarm optimization (PSO) algorithm is a nature-inspired optimization algorithm. It was developed by Kennedy and Eberhart (1995) [9], inspired from the natural behaviour of flocks of birds and schools of fish. PSO is a population-based stochastic algorithm used to solve SOPs based on the intelligent, coordinated movement of a swarm of particles. Multiobjective particle swarm optimization (MOPSO) is a variant of the PSO algorithm which is used to solve MOPs [10,11]. MOPSO has several advantages over PSO, including its ability to optimize multiple objectives, maintain diversity in the population, achieve better convergence to the true Pareto-optimal front, provide a range of solutions that represent the trade-off between the conflicting objectives, and being easy to implement. Moreover, the optimization accuracy of MOPSO is comparatively higher than a single-objective PSO.

Hence, this work adopts a simple, traditional approach to develop a CAD system for histopathology image detection and segmentation by combining the results from multilevel image thresholding and the superpixel algorithm. The optimal thresholds for multilevel histopathology image thresholding are obtained by modelling the thresholding problem as a MOP and solving the MOP using MOPSO. The reasons behind resorting to traditional approaches, such as thresholding and superpixel algorithms, for histopathology image segmentation are the following: (a) they are simple yet efficient, (b) they perform reasonably well even on small-to-medium-sized datasets, (c) the segmentation results produced by these algorithms are also comparable with several state-of-the-art methods, and (d) unlike deep learning models they do not require special hardware and also consume less time. Though thresholding is sensitive to grayscale inhomogeneities in the image, augmenting it with the result of the superpixel algorithm helps to achieve accurate segmentation. The superpixel algorithm is a simple, linear, iterative, clustering algorithm [12]. Superpixel algorithms are used in computer vision and image processing to group adjacent pixels into perceptually meaningful atomic regions. The resulting regions are typically more compact and uniform in colour and texture than individual pixels. Moreover, the superpixel algorithm is guided by the threshold value output by the multilevel thresholding algorithm to identify the correct ROIs. Thus, it could be seen that both the algorithms complement each other.

The proposed CAD system uses the multilevel multiobjective particle swarm optimization guided superpixel (MMPSO-S) algorithm. It consists of the following stages: pre-processing, segmentation, and post-processing. Initially, the RGB Hematoxylin and Eosin (H&E)-stained digital images from the histopathology image datasets are given as input to the system. In the pre-processing stage, the input images are first converted to grayscale and the contrast of the images is enhanced. The pre-processed images are then fed to the segmentation stage. This stage comprises two algorithms: (a) a multilevel thresholding algorithm called multilevel multiobjective particle swarm optimization (MMPSO), which uses the MOPSO algorithm and (b) a superpixel clustering algorithm which uses one of the thresholds obtained from the MMPSO algorithm to refine its output. The output segmentation maps from both the algorithms are combined together to provide the segmented ROIs. The final segmentation maps are then passed to the post-processing stage, which generates the final segmented image by eliminating the artifacts.

Stated below are the contributions of this work:

- The MMPSO algorithm with three different objective functions is used to identify the optimal threshold values for multilevel image thresholding. The MOPSO algorithm is applied for the first time in the field of histopathology image segmentation for multilevel image thresholding. This framework opens a new avenue for researchers to propose segmentation models which include more than one segmentation criterion. It should be noted that in the past, only the PSO algorithm with a single objective function has been used for the segmentation of nuclei regions from histopathology images;
- The proposed MMPSO-S algorithm combines the segmentation output of the MMPSO algorithm and the superpixel clustering algorithm; specifically, the threshold values obtained from the MMPSO algorithm are used to refine the output of the superpixel algorithm. This combined algorithm helps to improve the segmentation results;
- The proposed algorithm is applied to four different H&E-stained histopathology datasets for the detection and segmentation of various ROIs;
- The performance of the proposed method is compared with other single and multiobjective algorithms and also with the existing work performed on the datasets.

The rest of this paper is organized as follows. Section 2 describes the related works. Section 3 describes the datasets used for this work. The various stages of the proposed method are explained in Section 4. Section 5 discusses the experimental results obtained. The paper is concluded in the final segment Section 6.

## 2. Related Works

This section describes the related works in the field of image segmentation using PSO, multiobjective algorithms and superpixel algorithm.

### 2.1. Image Segmentation Using PSO and Its Variants

PSO is a population-based stochastic optimization algorithm that has been widely used for solving various optimization problems. In the context of image segmentation, PSO has been applied to find the optimal threshold values for segmentation. Various modifications to PSO have been proposed to improve its performance for image segmentation, such as Darwinian particle swarm optimization (DPSO) and fractional order Darwinian particle swarm optimization (FODPSO).

Jothi and Rajam [13] proposed a PSO-based Otsu’s multilevel thresholding method for the automatic segmentation of nuclei from the UCSB bio-segmentation dataset. Otsu’s thresholding was considered as an optimization problem. Precision, recall, and F-measure were used as the evaluation metrics, all of which had high values for the dataset. Liu et al. [14] proposed a PSO-based image clustering approach with intra-cluster distance as an optimization function. Breast cancer histopathology images with magnification levels 40×, 100×, 200×, and 400× were used for checking the effectiveness of the proposed approach. The experimental analysis showed that PSO performed better than the genetic algorithm (GA) and K-means.

A number of studies have been carried out using PSO for the segmentation of images in other fields. Chakraborty et al. [15] developed an improved PSO-based multilevel thresholding to identify the optimal thresholds. This algorithm was tested on some grayscale images and medical images other than histopathology images. It was found to provide better fitness value and lesser CPU time when compared to existing algorithms, such as modified artificial bee colony, cuckoo search, firefly, PSO, and GA. Another improved image segmentation method based on dynamic particle swarm optimization was proposed by Li et al. [16]. This algorithm was applied to a large set of real crystal growth images. The experimental results showed that the proposed algorithm can successfully separate the texture of crystal growth images and provide high robustness. A PSO-based multilevel thresholding using Kapur’s and Tsallis entropy was explored by Saini et al. [17]. This method was applied to normal brain magnetic resonance imaging (MRI). From the analysis, it was observed that Tsallis entropy worked more efficiently for the segmentation of cerebro spinal fluid and white matter regions when compared to Kapur’s entropy. Peng et al. [18] proposed an improved PSO-Fuzzy C-means (PSO-FCM) algorithm for the segmentation of images obtained from a standard image dataset. Experimental results showed that this clustering segmentation algorithm provides better accuracy and noise resistance.

DPSO and FODPSO are two variants of the PSO algorithm which have been used in the following studies for image segmentation. Suresh and Lal [19] proposed an improved variant of the DPSO algorithm based on chaotic functions to improve the convergence rate of DPSO and the segmentation quality of satellite images. The effectiveness of the model was compared with other optimization algorithms, such as cuckoo search, harmony search, differential evolution, and PSO. It was found that the algorithm suffered from higher computational complexity than the other algorithms. Tang et al. [20] applied the FODPSO algorithm for infrared image segmentation and defective edge recognition. The FODPSO algorithm helped to overcome the problem of high noise and fuzzy edges of the acquired infrared images. Guo et al. [21] developed a FODPSO algorithm for optic disc localisation and segmentation. The objective function used by the FODPSO algorithm was the between-class variance. The effectiveness of the algorithm was computed by experimenting on the retinal images from DRION, MESSIDOR, ORIGA, and other public databases.

### 2.2. Image Segmentation Using Multiobjective Algorithms

In recent years, researchers have explored the use of multiobjective optimization algorithms for image segmentation. Multiobjective optimization involves simultaneously optimizing multiple objectives, which in the context of image segmentation can correspond to different measures of segmentation quality, such as boundary adherence, region homogeneity, and compactness. By optimizing multiple objectives, multiobjective algorithms can produce diverse sets of solutions that can capture different trade-offs between segmentation criteria. Several multiobjective algorithms have been proposed for image segmentation, such as NSGA-II, MOEA/D, and MOGWO.

Zhe Liu [22] proposed an unsupervised image segmentation method using multiobjective PSO (UISMOPC) with two objective functions. This method was tested on the data obtained from the Berkeley segmentation dataset. From the experiments conducted, it was concluded that the UISMOPC algorithm is superior to the traditional K-means, FCM, and other clustering algorithms based on single objective functions. Maryam et al. [23] developed a MOPSO algorithm with two objective functions based on the entropy calculation of the image. This method provided good segmentation results when applied to some standard images. Hinojosa et al. [24] proposed a multiobjective colour thresholding method to reduce the overlapping effect on segmented images. This method was evaluated on the Berkeley image dataset and results showed that the multiobjective colour thresholding method provided better segmentation over traditional single-objective approaches by reducing overlapped areas on the image. A method for segmentation of human brain MRI using a multiobjective optimization approach based on fuzzy entropy clustering and region-based active contour was proposed by Pham et al. [25]. This algorithm was tested on simulated MRI and real MRI from the McConnell Brain Imaging Center (BrainWeb) and Internet Brain Segmentation Repository (IBSR). The proposed technique achieved superior segmentation performance in terms of accuracy and robustness. Elaziz et al. [26] proposed a multiobjective multiverse optimization algorithm for the segmentation of grayscale images. Kapur and Otsu were the two objective functions used. This method was tested on 11 natural grayscale images and was found to provide a better pareto optimal front than other algorithms in terms of hypervolume and spacing. Multiobjective grey wolf optimization (MOGWO), an extension of the grey wolf optimization algorithm was introduced by Oliva et al. [27]. Experiments were conducted using this algorithm on a set of popular natural grayscale images by calculating performance metrics such as PSNR, SSIM, fitness function, and CPU time. The MOGWO based on Kapur and Otsu functions achieved better segmentation results compared to other existing algorithms. Another image segmentation method based on multiobjective artificial bee colony optimization was introduced by Sag and Cunkas [28]. This method was applied to several natural images obtained from the Berkeley segmentation database. The segmentation results obtained from this method were found to be better than FCM.

### 2.3. Image Segmentation Using Superpixel Algorithm

Superpixels are a group of pixels that share similar properties, such as colour or texture. Superpixel-based segmentation has become increasingly popular in recent years due to its ability to provide a more compact representation of an image and improve the accuracy of segmentation. In most of the studies, a superpixel algorithm combined with other segmentation algorithms was found to improve segmentation accuracy.

Albayrak Abdulkadir [29] proposed a simple linear iterative clustering (SLIC) superpixel segmentation method and convolutional neural network (CNN) method to segment cells from histopathology images. This method had two stages: firstly, a pre-segmentation was performed using a SLIC superpixel method and then a CNN-based deep learning method was used to classify those superpixels to obtain the final segmentation. The performance of the method was tested on kidney renal cell carcinoma histopathological images of The Cancer Genome Atlas (TCGA) data portal. An overall accuracy of 0.98 was obtained. Albayrak and Bilgin [30] proposed a two-staged superpixel algorithm for the segmentation of cells from histopathology images. In the first stage, the images were segmented using the SLIC method and then the superpixels were clustered using clustering-based segmentation algorithms. The performance of this algorithm was tested on high-resolution histopathological images of renal cell carcinoma, selected from the TCGA data portal. Ding et al. [31] proposed an image segmentation algorithm based on superpixel clustering. In the first step, the images were divided into a set of superpixels using superpixel pre-processing techniques. Next, a spectral clustering algorithm was applied to cluster the superpixel regions and to obtain the final segmented image. This algorithm was tested on the satellite images from the UC Merced Land Use Dataset and the experimental results showed that this algorithm gave a better performance over other traditional spectral clustering algorithms. Zhang et al. [32] proposed a method based on the superpixel and expectation maximization (EM) algorithms for the segmentation of leaves with plant diseases. Firstly, the superpixel algorithm divided the images into several superpixels, and then the EM algorithm was applied to segment the lesion pixels from the image. Experimental results showed that the proposed method was appropriate for plant disease leaf image segmentation.

Table 1 gives a summary of the related works in image segmentation using PSO, multiobjective algorithms, and superpixel algorithm. From the table, it is clear that PSO and its variants are used for image segmentation, but very little work has been carried out on PSO and its variants on histopathology segmentation. It can be noted that MOPSO has never been applied to histopathology image segmentation. The superpixel algorithm is found to improve the segmentation accuracy of other segmentation algorithms. In this paper, we propose a multilevel multiobjective PSO-guided superpixel algorithm to segment ROIs from histopathology images.

## 3. Dataset Description

**Figure 1:**
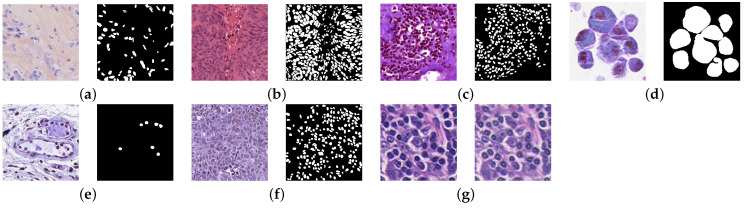
Sample images and their corresponding masks from the datasets: (**a**) TNBC. (**b**) MoNuSeg. (**c**) MoNuSAC-L. (**d**) MoNuSAC-M. (**e**) MoNuSAC-N. (**f**) MoNuSAC-E. (**g**) LD.

The effectiveness of the proposed algorithm was tested on four different histopathology image datasets used for the segmentation and detection task. Table 2 gives the summary of the datasets used in this work. Sample images from the datasets along with their corresponding masks are given in Figure 1. A detailed explanation of the datasets is given below:

### 3.1. Triple-Negative Breast Cancer Dataset

The triple-negative breast cancer (TNBC) dataset contains 50 H&E-stained breast histopathology images and their corresponding masks, each of dimension 512 × 512. All images are in .png format. There are 4022 annotated cell nuclei in the dataset. Segmentation of nuclei cells is the task to be performed [33].

### 3.2. Multi-Organ Nuclei Segmentation Dataset

The multi-organ nuclei segmentation (MoNuSeg) dataset contains H&E-stained histopathology images from 30 patients with tumours of liver, kidney, prostate, bladder, breast, colon, and stomach organs, captured at 40× magnification [34,35]. The dataset has 44 images containing 29,000 nuclear boundary annotations. Each image is of size 1000 × 1000. Segmentation of nuclei cells is the task to be performed on the dataset.

### 3.3. Multi-Organ Nuclei Segmentation and Classification Dataset

The multi-organ nuclei segmentation and classification (MoNuSAC) dataset contains H&E-stained tissue images of four organs: lungs, prostate, kidney, and breast. The images have lymphocytes, macrophages, epithelial cells, and neutrophils. All images are of type .tif containing 31,000 nuclear boundaries. The task for this dataset is the segmentation of lymphocytes (L), macrophages (M), neutrophils (N), and epithelial cells (E). From the MoNuSAC dataset, four sub-datasets are created for each task denoted as MoNuSAC-L, MoNuSAC-M, MoNuSAC-N, and MoNuSAC-E [36].

### 3.4. Lymphocyte Detection Dataset

The lymphocyte detection (LD) dataset consists of 100 H&E-stained ER + BCa images scanned at 20× magnification. Each image has a dimension of 100 × 100 and is in .tif format. The centres of 3064 lymphocytes were identified by an expert pathologist. The task is the detection of lymphocyte centres in the images [37].

## 4. The Proposed Method

**Figure 2:**
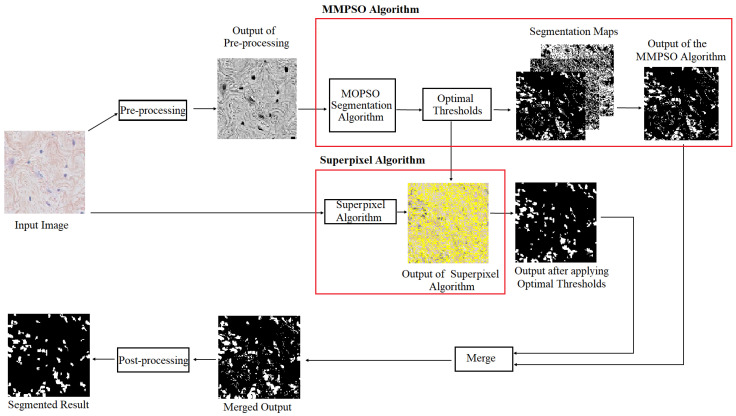
General representation of the proposed CAD system.

The proposed CAD system uses a multilevel multiobjective particle swarm optimization guided superpixel algorithm (MMPSO-S) for the efficient detection and segmentation of ROIs from histopathology images. Figure 2 shows the steps involved in the proposed system. It consists of two different pipelines for processing the input image.

The first pipeline is the MMPSO algorithm, where the RGB histopathology image is pre-processed and then passed to the MOPSO algorithm to generate optimal threshold values. The optimal thresholds generated by the MOPSO algorithm are applied to the input image, which generates three segmentation maps. The segmentation map containing the ROIs is selected for further processing. This is the output of the MMPSO algorithm.

In the second pipeline, the RGB histopathology image is passed to the superpixel algorithm to generate superpixels. The superpixel algorithm helps to identify the ROIs with the proper boundary. Optimal thresholds generated by the MMPSO algorithm are used to refine the output obtained from the superpixel algorithm.

The output images from the superpixel algorithm and the MMPSO algorithm are combined to generate the final output image. Finally, post-processing of the image is performed to improve the quality of segmentation. A detailed explanation of each step is given in the following subsections.

### 4.1. Pre-Processing

The pre-processing stage prepares the acquired histopathology image for segmentation. Histopathology images undergo different stages of slide preparation and may be affected by noise, blur, and poor contrast, which can lead to inaccurate diagnosis. It is essential to eliminate the noise and artifacts and enhance the image quality to obtain accurate ROIs. Pre-processing of images helps to increase the quality of the image and reduce the complexity of further processing [38].

**Figure 3:**
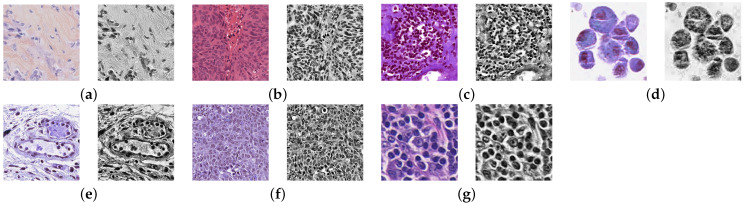
Sample images and corresponding pre-processed images from the datasets: (**a**) TNBC. (**b**) MoNuSeg. (**c**) MoNuSAC-L. (**d**) MoNuSAC-M. (**e**) MoNuSAC-N. (**f**) MoNuSAC-E. (**g**) LD.

In this work, the RGB images were first converted to grayscale images. The contrast of the grayscale images was then enhanced by applying the contrast-limited adaptive histogram equalization (CLAHE) method [39]. Figure 3 shows sample images from each dataset before and after pre-processing.

### 4.2. MMPSO-S Algorithm for Detection and Extraction of ROIs

This section explains the process of separating the ROIs from histopathology images using the MMPSO-S algorithm. A pre-processed histopathology image $I_{pre}$ is given as the input to the MMPSO-S algorithm, and a segmented image $I_{merge}$ is obtained as the output. The working of the MMPSO-S algorithm is explained in the following subsections.

#### 4.2.1. MMPSO for Multilevel Image Thresholding

The MMPSO algorithm uses the MOPSO algorithm, which is a population-based algorithm to solve MOPs. The MOPSO algorithm uses an external memory and a geographically based approach to maintain diversity. It has an initialization phase and an iterative phase. This section provides the MMPSO algorithm for multilevel image thresholding.

**KeyTerms of MOPSO Algorithm**

- Decision space: Decision space/search space is the vector space of all decision variables. The search space varies depending on the problem domain.
- Objective space: Objective space is the vector space of all solutions obtained from the evaluation of the decision variables.
- Particles and swarm: Swarm is a collection of particles. Particles are individuals, such as birds or fishes, in the swarm. Let *i* represent a particle in the swarm and *i* = 1, 2, …, $N_{par}$, where $N_{par}$ is the population size.
- Position: Each particle *i* in the search space has two properties, i.e., position and velocity. The position of a particle *i* is denoted as $X_{i}$ and is considered as the feasible solution to the optimization problem. It has upper and lower limits, which are the boundary of the search space denoted as $\left[X_{min},X_{max}\right]$.
- Velocity: Velocity of a particle $V_{i}$ defines its ability to move in the search space, which allows the particle to update its position. The upper and the lower limits of the velocity are denoted as $\left[V_{min},V_{max}\right]$.
- Objective function: It is also known as the fitness function/cost function. The objective function maps an element from the decision space to the objective space. The objective function is evaluated using the position $X_{i}$ and the outcome is a real number known as the cost value or the fitness value. In the case of MOPSO, the outcome of all objective functions form a vector.
- Local best: The local best value for a particle is the position value which gives the best fitness value in the whole history of its movement. It is denoted by $pBest_{i}$.
- Feasible solution set: A solution that satisfies all the constraints of an MOP is called a feasible solution. A set of all feasible solutions is called the feasible solution set.
- Non-dominated solution: A feasible solution is non-dominated if there does not exist another feasible solution better than the current one in some objective function without worsening another objective function.
- External repository: It is a storage space to store all the best particles (non-dominated solutions) [10]. This repository is often known as an external archive and is denoted by *A*. External repository has a maximum size ($A_{max}$). To avoid the high computational cost of searching and updating the external repository, its size is limited.
- Leader: From the external repository, one solution (*L*) is selected as the leader for the entire swarm and its position is taken as the $pBest_{L}$ value.

**Initialization Phase of MOPSO Algorithm**

The swarm, its particles, and other parameters of the MOPSO algorithm are initialized according to the image segmentation problem. The intensity range of the histopathology grayscale image [0,255] is treated as the search space. The position of each particle *i* in the swarm is represented as $X_{i}=\left(x_{1},x_{2}\right)$, where initially $x_{1}$ and $x_{2}$ are the two random intensity values within the range of [0, 255]. The velocity range is set to [−5, 5]. Initially, the velocity $V_{i}$ of all particles is set to zero. In this work, the number of particles ($N_{par}$), the number of iterations ($N_{ite}$), and the number of thresholds (*k*) are set to 150, 150, and 2, respectively, after experimental analysis. The external archive is initially empty and its size is fixed to 30. The initial parameters of the MOPSO algorithm used in this work are shown in Table 3.

After initializing the parameters of the MOPSO algorithm, the fitness values of each particle are calculated for all the fitness functions. In this work, three different objective functions, Otsu’s method [40], Kapur’s entropy [41], and Renyi’s entropy [42], were used to find the optimal threshold values. The personal best of each particle ($pBest_{i}$) is initially equal to the position of the particle ($X_{i}$). The non-dominated solutions are then identified and stored in the external repository.

**Objective Functions**

This section details the three objective functions used in this work. Let $n_{i}$ be the number of pixels in the intensity level *i* and np be the total number of pixels in the image, then the probability of intensity level *i* can be defined as $P_{i}=\frac{n_{i}}{np}$. The *k* thresholds, $t_{1},t_{2},\ldots ,t_{k}$, divide the image into k+1 regions denoted as $R_{1},R_{2},\ldots ,R_{k+1}$. $\mu_{T}$ is the mean intensity of the whole image and is given by Equation (1). $\mu_{i}$ is the mean intensity of the region *i* and is given by Equation (2). $\omega_{i}$ is the probability distribution of the region *i* and is given by Equation (3).


(1) $$\mu_{T}=\sum_{i=0}^{255}iP_{i}$$

(2) $$\mu_{1}=\sum_{i=0}^{t_{1}-1}\frac{iP_{i}}{\omega_{0}},\;\;\mu_{2}=\sum_{i=t_{1}}^{t_{2}-1}\frac{iP_{i}}{\omega_{1}},\;\;\ldots ,\;\;\mu_{k+1}=\sum_{i=t_{k}}^{255}\frac{iP_{i}}{\omega_{k}}$$

(3) $$\omega_{1}=\sum_{i=0}^{t_{1}-1}P_{i},\;\;\omega_{2}=\sum_{i=t_{1}}^{t_{2}-1}P_{i},\;\;\ldots ,\;\;\omega_{k+1}=\sum_{i=t_{k}}^{255}P_{i}$$

(a) Otsu’s multilevel thresholding: Otsu’s method is an unsupervised and non-parametric threshold selection method [40]. In Otsu’s method, the threshold is selected by the discriminant criterion, that is to maximize the between-class variance among segmented regions/classes [43]. Otsu’s objective function ($f_{1}$) for the multilevel grayscale image segmentation is given by Equation (4).
  (4) $$f_{1}\left(t_{1},t_{2},\ldots ,t_{k}\right)=\sum_{i=1}^{k+1}\omega_{i}{\left(\mu_{i}-\mu_{T}\right)}^{2}$$
(b) Kapur’s multilevel thresholding: Kapur’s entropy is a generalization of Shannon’s entropy. In Kapur’s method, the threshold is selected by the discriminant criterion, that is to maximize the between-class entropy [41,44]. Kapur’s objective function ($f_{2}$) for the multilevel segmentation of grayscale images is given by Equation (5).
  (5) $$f_{2}\left(t_{1},t_{2},\ldots ,t_{k}\right)=KH_{1}+KH_{2}+\ldots +KH_{k+1},$$
  where $KH_{i}$ is the Kapur’s entropy of the region *i* and is given by Equation (6).
  (6) $$KH_{1}=-\sum_{i=0}^{t_{1}-1}\frac{P_{i}}{\omega_{0}}\ln\frac{P_{i}}{\omega_{0}},\;\;KH_{2}=-\sum_{i=t_{1}}^{t_{2}-1}\frac{P_{i}}{\omega_{1}}\ln\frac{P_{i}}{\omega_{1}},\;\;\ldots ,\;\;KH_{k+1}=-\sum_{i=t_{k}}^{255}\frac{P_{i}}{\omega_{k}}\ln\frac{P_{i}}{\omega_{k}}$$
(c) Renyi’s multilevel thresholding: Renyi’s entropy is a generalized form of Shannon’s entropy with a parameter α used to evaluate the randomness of a system. When α = 1, Renyi’s entropy is equal to Shannon’s entropy [42]. Renyi’s objective function ($f_{3}$) for the multilevel segmentation of grayscale images is given by Equation (7).
  (7) $$f_{3}\left(t_{1},t_{2},\ldots ,t_{k}\right)=RH_{1}+RH_{2}+\ldots +RH_{k+1},$$
  where $RH_{i}$ is the Renyi’s entropy of the region *i* and is given by Equation (8).
  (8) $$RH_{1}=\frac{1}{1-\alpha }\ln\sum_{i=0}^{t_{1}-1}{\left(\frac{P_{i}}{\omega_{0}}\right)}^{\alpha },\ldots ,\;\;RH_{k+1}=\frac{1}{1-\alpha }\ln\sum_{i=t_{k}}^{255}{\left(\frac{P_{i}}{\omega_{k}}\right)}^{\alpha }$$
  For better segmented output, the above objective functions must be maximized.

**Iterative Phase of MOPSO Algorithm**

After the initialization phase, the iterative phase is executed for a specified number of iterations ($N_{ite}$). During each iteration, particles in the swarm and external archive are updated. The steps involved in the iterative phase are given below:

1. **Leader selection from the external archive:**
  The repository with the non-dominated solutions is mapped to an adaptive grid with a grid size $G_{size}$ comprising hypercubes [10]. Each non-dominated solution from the archive is placed in the hypercube by considering its fitness values as the coordinates. A hypercube can hold ns number of non-dominated solutions where ns>1. The following steps are used to select a leader from the non-dominated solutions:
  (a) The fitness value of a hypercube is calculated by dividing any number *x* (x> 1) by the number of particles in that hypercube.
  (b) A roulette wheel algorithm is used to select a hypercube using the fitness values.
  (c) If the selected hypercube has one particle, then the particle is set as the leader of the swarm. Otherwise, if the number of particles in the selected hypercube is greater than 1 (i.e., ns>1), then one particle is chosen randomly and is set as the leader of the swarm.
2. **Update position and velocity of each particle:**
  Once the leader is selected, the velocity and position of all the particles in the swarm are updated using Equations (9) and (10).
  (9) $$V_{i}\left(t+1\right)=\omega V_{i}\left(t\right)+c_{1}r_{1}\left(X_{i}\left(t\right)-pBest_{i}\left(t\right)\right)+c_{2}r_{2}\left(X_{i}\left(t\right)-pBest_{L}\left(t\right)\right)$$
  (10) $$X_{i}\left(t+1\right)=X_{i}\left(t\right)+V_{i}\left(t+1\right),$$
  where ω is known as the inertia parameter, $X_{i}\left(t\right)$ is the position of the particle *i* at time *t* and $V_{i}\left(t\right)$ is the velocity of the particle *i* at time *t*, $X_{i}\left(t+1\right)$ is the position of the particle *i* at time t+1, and $V_{i}\left(t+1\right)$ is the velocity of the particle *i* at time t+1. $c_{1},c_{2}$ are the positive constants known as acceleration coefficients. $r_{1},r_{2}$ are the random numbers in the range (0, 1). In this work, $\omega ,c_{1},c_{2}$ are set to 1.3, 0.5, and 0.5, respectively.
3. **Compute fitness values for each particle:**
  Once the position value of each particle *i* in the swarm is updated, the fitness values of each particle are calculated for all the fitness functions.
4. **Update the local best value of each particle:**
  If the current $pBest_{i}$ value of a particle *i* is dominated by the new position value $X_{i}\left(t+1\right)$ of the particle, then the current $pBest_{i}$ value of the particle is replaced with the $X_{i}\left(t+1\right)$ value. Otherwise, the current $pBest_{i}$ value of the particle *i* is kept as it is. If neither the current $pBest_{i}$ value nor the new position value of a particle are dominating each other, then one of the values is randomly selected as the $pBest_{i}$.
5. **Update the external repository:**
  The non-dominated particles are identified based on the pareto dominance condition [10]. The non-dominated particles are compared with the particles already existing in the external archive in order to decide their inclusion to the external archive. The MOPSO algorithm follows four rules to add a non-dominated particle to the archive:
  (a) If the archive is empty, then the new particle is added to the archive.
  (b) If the particle is dominated by any of the particles in the archive, then the new particle is discarded.
  (c) If none of the particles in the archive dominate the new particle, and if the archive has enough space, then the new particle is added to the archive. During the entry, any particle in the archive dominated by the new particle is deleted from the archive.
  (d) If none of the particles in the archive dominate the new particle and the archive does not have enough space, then the particle from the most crowded hypercube is removed and the new particle is inserted in the archive. During the time of entry, any particle in the archive that is dominated by the new particle is removed from the archive.
6. **Apply mutation operator to the particles:**
  The relevance of the mutation operator in the MOPSO algorithm is to allow the algorithm to explore the search space with a high exploratory capability. During the initial iterations of the algorithm, the mutation operator affects all the particles in the search space; however, the number of particles affected by the operator decreases as the number of iterations increases. In this work, the mutation rate (μ) is set to 0.1.

At the end of the algorithm’s execution, the external repository contains the best/non-dominated particles from the swarm.

**Obtaining the Optimal Threshold Values**

To obtain the optimal threshold values, the best particle is selected from the external archive *A* based on the euclidean distance measure [45]. The euclidean distance method is one of the most simple and straightforward method that works well on low-dimensional data. At first, the euclidean distance of every particle in *A* from the origin of the objective space is calculated. The particle having the highest euclidean distance (denoted as BestParticle) is then selected for further processing. Let *i* be a particle having three fitness values $f_{1},f_{2}$, and $f_{3}$, and O(0,0,0) denote the origin of the objective space, then the euclidean distance between the origin and particle *i*, denoted as d(O,i), is given by Equation (11).

(11) $$d\left(O,i\right)=\sqrt{{\left(f_{1}\right)}^{2}+{\left(f_{2}\right)}^{2}+{\left(f_{3}\right)}^{2}}$$

The position ($x_{1},x_{2}$) of the BestParticle having the highest euclidean distance is taken as the optimal threshold values $t_{1}$ and $t_{2}$ (i.e., $t_{1}=x_{1}$ and $t_{2}=x_{2}$). $x_{1}$ and $x_{2}$ are the best intensity values of the corresponding input image for multilevel thresholding.

**Generating segmentation maps:**

**Figure 4:**
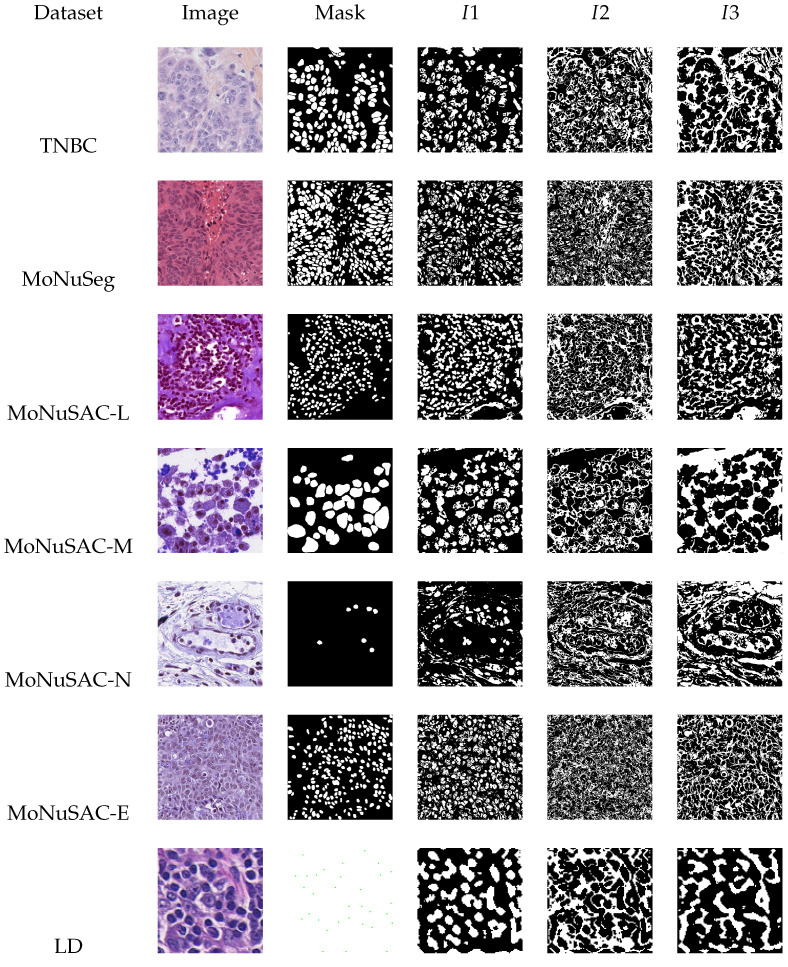
Sample images, ground truth masks, and segmentation maps generated after applying threshold values.

The two threshold values obtained from the MOPSO algorithm are used to partition the pre-processed image, $I_{pre}$, into three binary images. The first image (I1) contains pixels whose intensity values fall in the range of [0, $t_{1}-1$], the second image (I2) contains pixels having intensity values in the range of [$t_{1}$, $t_{2}-1$], and the third (I3) image contains pixels whose intensity values fall in the range of [$t_{2}$, 255]. From the three images, image I1 containing the ROIs is chosen for post-processing. The other two images I2 and I3 do not include the ROIs and hence are not considered. Figure 4 shows the sample images along with their corresponding ground truth and segmentation maps I1, I2, I3 after applying threshold values $t_{1}$ and $t_{2}$.

#### 4.2.2. Segmentation by Superpixel Algorithm

The superpixel algorithm is a simple, linear, iterative, clustering algorithm [12]. A group of pixels having common characteristics is called a superpixel/cluster. In this work, the RGB colour histopathology image $I_{color}$ is passed to the superpixel algorithm as input. The output is a label matrix representing superpixels C_1_, C_2_, … , C$nc^{{}^{\prime }}$ where $nc^{{}^{\prime }}$ is the actual number of superpixels generated [46,47].

Let the input image to the superpixel algorithm have np number of pixels, and nc be the expected number of superpixels to be generated. Then, the total number of pixels in each superpixel is np/nc. A pixel in the input image can be denoted as [$R_{i},G_{i},B_{i},X_{i},Y_{i}$] and 1<i<np. $R_{i},G_{i}$, $B_{i}$, $X_{i}$, and $Y_{i}$ denote the RGB colour component and X and Y coordinates of the *i*-th pixel [46]. The centre of a superpixel is defined as the mean value of all the pixels in the superpixel. The average distance (*S*) between the centres of two nearby superpixels is $\sqrt{np/nc}$.

The algorithm works as follows: A grid with distance between the grid lines as *S* is initially placed over the image. The intersection points of the grid lines denote the initial cluster centres $C_{1},C_{2},\ldots ,C_{nc}$. Then, for each pixel in the image, the dissimilarity between the pixel and the cluster centres in its 2S×2S neighbourhood is found according to the distance measure given by Equation (12).

(12) $$\begin{array}{cc} \; & d_{RGB}=\sqrt{{\left(R_{i}-R_{j}\right)}^{2}+{\left(G_{i}-G_{j}\right)}^{2}+{\left(B_{i}-B_{j}\right)}^{2}}\\ \; & d_{XY}=\sqrt{{\left(X_{i}-X_{j}\right)}^{2}+{\left(Y_{i}-Y_{j}\right)}^{2}}\\ \; & D_{m}=d_{RGB}+\frac{p}{S}d_{XY}, \end{array}$$

where $d_{RGB}$ represents the distance of colour values of the pixel *i* and cluster centre *j*, $d_{XY}$ represents the Euclidean distance or spatial distance between the pixel *i* and cluster centre *j*, $D_{m}$ is the final distance. A variable *p* is used while computing $D_{m}$ to control the compactness of a cluster. Higher compactness is obtained for higher values of *p*. A pixel is assigned to the cluster centre with the least distance.

After processing all pixels in the image, the new cluster centres are identified. The residual error *E* is computed as the sum of the differences between the new cluster centre and the previous cluster centre. The algorithm is repeated until the residual error *E* falls below a threshold value. The final output image is $I_{super}$ with $nc^{{}^{\prime }}$ number of clusters, where $nc^{{}^{\prime }}\leq nc$. For this work, we choose nc as 1000 because a WSI contains more pixels than normal images, and the ROIs in a WSI are very small.

**Refining the clusters:**

The clusters output by the superpixel algorithm sometimes may not be the desired ROIs. Hence, in order to improve the segmentation output by the superpixel algorithm, the clusters are refined using the threshold obtained from the MMPSO algorithm. Since the ROIs in the images have a pixel intensity value that is less than t1 (obtained from MMPSO), we use this threshold value to further refine the clusters obtained from the superpixel algorithm. For this, we use an empty image $I_{thresh}$ with the same dimensions as $I_{super}$, initially consisting of all zeros. Clusters in $I_{super}$ whose average pixel intensity value is less than the threshold value $t_{1}$ are identified, and the pixels corresponding to those clusters are added to $I_{thresh}$. Thus, $I_{thresh}$ contains all clusters in $I_{super}$, whose pixel intensity value is less than $t_{1}$.

### 4.3. Combining Segmentation Maps and Post-Processing

The segmentation map I1 from the MMPSO algorithm and output from the superpixel algorithm $I_{thresh}$ are combined to form a single image $I_{merge}$. This combination of images reduces the ROIs’ border irregularities and helps to find the exact area of the ROIs. The $I_{merge}$ is given as an input to the post-processing phase. The post-processing stage improves the segmented image by eliminating the artifacts in it.

**Figure 5:**
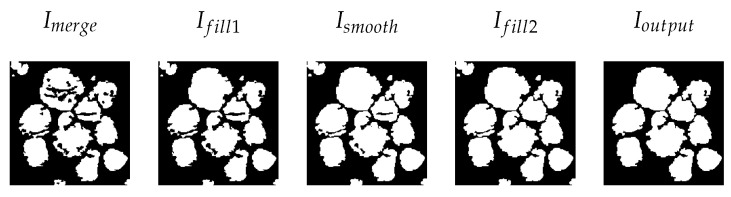
Post-processing of a sample image from dataset.

In this work, the post-processing methods, such as hole filling, edge smoothing, and removing small ROIs, are applied to the segmented image to increase the segmentation accuracy. Figure 5 shows a sample image before and after post-processing. Histopathology image segmentation using the MMPSO-S algorithm is given in Algorithms 1 and 2.
**Algorithm 1:** MMPSO algorithm.**Input:** Pre-processed image $I_{pre}$**Output:** Segmentation map $I_{1}$**Parameters:** MMPSO parameters, i.e., $N_{par}$, $N_{ite}$, *k*, $X_{min}$, $X_{max}$, $V_{min}$, $V_{max}$ and $A_{max}$initialize all MMPSO parameters with the values presented in Table 3**for** each particle i=1 to $N_{par}$ **do**    randomly assign position $X_{i}$ within the permissible range    initialize velocity, $V_{i}$ = 0    compute fitness values of the particle    initialize local best, $pBest_{i}=X_{i}$**end for**identify non-dominated solutionsstore all non-dominated solutions in external repository, *A*initialize variable t = 1**while** $t<N_{ite}$ **do**    **for** each particle i=1 to $N_{par}$ **do**        select a leader from *A*        compute velocity $V_{i}$ using Equation (9)        compute position $X_{i}$ using Equation (10)        compute fitness values of the particle        update $pBest_{i}$    **end for**    update repository *A* with new non-dominated solutions    apply mutation operator**end while**initialize variable max=0**for** each particle i∈A **do**    temp←d(O,i)    **if** temp>max **then**        max←temp        BestParticle←i    **end if****end for**$\left[t_{1},t_{2}\right]\leftarrow BestParticle\left(x_{1},x_{2}\right)$initialize images I1, I2, I3 with the same size as $I_{pre}$ and all pixel values as zero**for** each pixel $i\in I_{pre}$ **do**    $Inten_{i}\leftarrow$ intensity of pixel *i*    **if** ($0\leq Inten_{i}<t_{1}$) **then**        I1(i)← 1, I2(i)← 0, I3(i)← 0    **else if** ($t_{1}\leq Inten_{i}<t_{2}$) **then**        I1(i)← 0, I2(i)← 1, I3(i)← 0    **else** ($t_{2}\leq Inten_{i}<255$)        I1(i)← 0, I2(i)← 0, I3(i)← 1    **end if****end for**return I1

**Algorithm 2:** Superpixel algorithm, cluster refinement, output merging, and post-processing.
**Input:** Histopathology colour image, $I_{color}$ and output image from MOPSO algorithm I1
**Output:** Image after post-processing $I_{output}$
**Parameters:** Expected number of superpixels to be generated nc, Cluster centres $C_{1},C_{2},\ldots ,C_{nc}$
initialize cluster centres $C_{1},C_{2},\ldots ,C_{nc}$
**do**
    **for** each pixel **do**        find the distance of the pixel with the cluster centres in its 2S × 2S neighbourhood according to the distance measure using Equation (12)        assign the pixel to the centre with which it has least distance    **end for**    compute new cluster centres    compute the error *E* as the difference between new cluster centre and previous centre**while** E> threshold$I_{super}\leftarrow$ output of superpixel algorithm**for** each cluster **do**    AvgC ← Average pixel intensity of the cluster    **if** AvgC $<t_{1}$ **then**        $I_{thresh}\leftarrow I_{thresh}\cup$ cluster    **else**        Discard the cluster    **end if**
**end for**
$I_{merge}\leftarrow$ merge I1 and $I_{thresh}$**for** each image $I_{merge}$ in the dataset **do**    $I_{fill}\leftarrow$ fill holes in the ROIs from $I_{merge}$    $I_{smooth}\leftarrow$ smooth the edges of ROIs from $I_{fill}$    $I_{output}\leftarrow$ remove small ROIs from $I_{smooth}$
**end for**
Return $I_{output}$


## 5. Results and Discussion

In this section, we investigate the applicability of the proposed MMPSO-S algorithm in histopathology image detection and segmentation. The experimental analysis of the proposed MMPSO-S algorithm has been studied in three subsections. Section 5.1 gives the tuning of the MMPSO-S algorithm parameters. Section 5.2 presents the segmentation performance of the proposed algorithm over other single objective and multiobjective algorithms. The normalised execution time per 1 megapixel of the segmentation algorithms are given in Section 5.3.

### 5.1. Parameter Tuning

The values of the parameters for the MMPSO-S algorithms were determined through empirical analysis. The parameters are population size ($N_{par}$), number of iterations ($N_{ite}$), external repository size ($A_{max}$), and grid size ($G_{size}$). A grid search was conducted to find the best values for the parameters. The grid search was conducted by varying the population size as 50, 75, 100, 125, 150, and 175, the number of iterations as 50, 75, 100, 125, 150, and 175, the external archive size as 30, 60, and 100, and the adaptive grid size as 7, 20, 30, and 40. A total of 10 images from each dataset (TNBC, MoNuSeg, MoNuSAC-L, MoNuSAC-M, MoNuSAC-N, MoNuSAC-E, and LD) were randomly selected and a total of 70 images were obtained. The fitness values for each image were calculated by varying the parameter values within certain intervals. The parameter values that give the best fitness values were chosen. From the experimental results, it is noted that high fitness values for $f_{1}$, $f_{2}$ and $f_{3}$ are observed; $N_{par}$ is 150, $N_{ite}$ is 150, $A_{max}$ is 30, and $G_{size}$ is 7.

**Figure 6:**
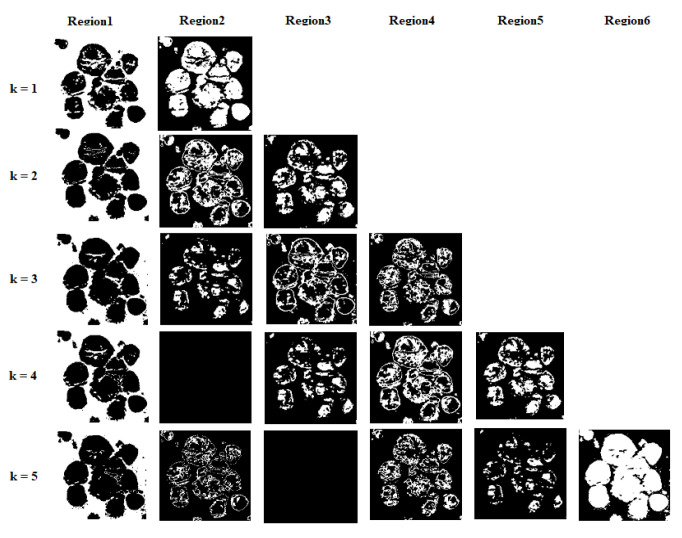
The output images of the MMPSO segmentation algorithm when the number of thresholds is varied as 1, 2, 3, 4, and 5.

Another experiment was conducted for setting the value of the number of thresholds (*k*) by varying *k* as 2, 3, 4, and 5. Figure 6 given below shows the output image of the MMPSO segmentation algorithm when the number of thresholds is varied as 1, 2, 3, 4, and 5. From the experiments, it is observed that when the number of thresholds increased, the size of the ROIs (nuclei regions) was reduced in the segmented output. Conversely, when the number of thresholds decreased, the size of the nuclei regions increased beyond their actual size in the segmented image. For example, if the threshold is set to 5, the output will consist of six segmented images, each containing different regions of the original image, none of which will contain nuclei regions with perfect size. If the threshold is 1 then, we obtain two segmented images. These nuclei regions in the images have sizes larger than the actual size of the nuclei. If the threshold is 2, then we obtain three segmented images containing different regions. From these three images, we observed that the first image always contains nuclei regions of approximately the same size as the actual nuclei. So, we chose 2 as the proper number of thresholds. Additionally, we used the best parameter values for the multiobjective evolutionary algorithm based on decomposition (MOEA/D) [48] and the non-dominated sorting genetic algorithm 2 (NSGA-2) [49].

### 5.2. Segmentation Performance

To establish the superiority of the proposed method, it was compared with single-objective and multiobjective optimization algorithms. The single-objective algorithms are the PSO algorithm, the DPSO algorithm [50], and the FODPSO [51] algorithm. The multiobjective algorithms are the multiobjective PSO algorithm (MOPSO), MOEA/D [48], and NSGA-2 [49].

For the single-objective PSO, DPSO, and FODPSO algorithms, the objective function used is the Otsu’s discriminant criterion. For the MOPSO algorithm, we experimented with the combination of two and three objective functions. The objective functions used were Otsu’s discriminant criterion, Kapur’s entropy, and Renyi’s entropy. The various multiobjective experiments conducted by us are denoted as follows: MOPSO(Kapur + Otsu) is the multiobjective PSO algorithm with Kapur’s entropy and Otsu’s discriminant criterion as the objective functions. MOPSO(Kapur + Renyi) indicates the multiobjective PSO algorithm with Kapur’s entropy and Renyi’s entropy as the objective functions. MOPSO(Otsu + Renyi) is the multiobjective PSO algorithm with Otsu’s discriminant criterion and Renyi’s entropy as the objective functions. MOPSO(Otsu + Renyi + Kapur) is a multiobjective PSO algorithm with Otsu’s discriminant criterion, Renyi’s entropy, and Kapur’s entropy as the objective functions.

**Figure 7:**
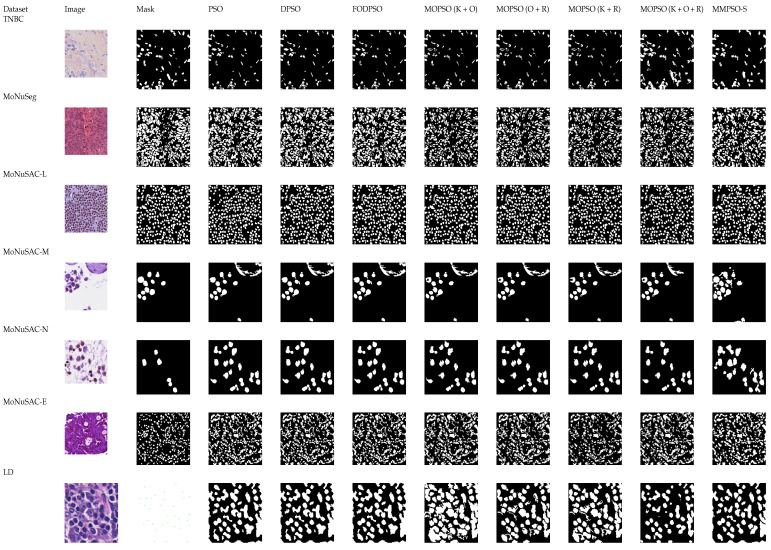
Final segmentation map of one sample image from datasets.

The proposed algorithm, MMPSO-S, is a combination of MMPSO with all three objective functions and the superpixel algorithm. The MOEA/D and NSGA-2 algorithms are implemented using three objective functions: Otsu’s discriminant criterion, Renyi’s entropy, and Kapur’s entropy. The final segmentation outputs of a sample image from each dataset are given in Figure 7. The evaluation metrics used for segmentation performance are F-measure, dice coefficient, and Jaccard coefficient. The evaluation metrics used for detection performance are recall, precision, and F-measure. The description of the evaluation metrics used in this work is given in Appendix A. Segmentation results obtained for all these algorithms are given in Tables 5–7.

**Segmentation Performance on the MoNuSeg Dataset:**

Table 4 shows the segmentation results of algorithms applied on the MoNuSeg dataset. The table also shows the results of the previous works on the MoNuSeg datasets using traditional segmentation methods. To the best of our knowledge, the previous studies carried out on the MoNuSeg dataset using traditional segmentation methods used Otsu threshold, watershed transform, Fiji, region growing, and active contour methods [52,53]. The table shows that the proposed MMPSO-S algorithm gives a high Jaccard value of 0.56, dice value of 0.72, and an F-measure of 0.72. The MOEA/D algorithm and other traditional segmentation methods give a very low Jaccard value. The NSGA2 algorithm provides a Jaccard value of 0.43 and a dice value of 0.58.

**Segmentation Performance on the TNBC Dataset:**

Table 5 shows the segmentation results of algorithms applied on the TNBC dataset. From the table, it is observed that the proposed MMPSO-S algorithm gives a high Jaccard value of 0.49, a dice value of 0.65, and an F-measure value of 0.65. The segmentation results given by the MOEA/D algorithm are comparatively very low. The NSGA2 algorithm gives a Jaccard value of 0.42 and a dice value of 0.58.

**Segmentation performance on the MoNuSAC dataset:**

Segmentation results of the MoNuSAC dataset are given in Table 6. For the lymphocyte segmentation dataset, the MMPSO-S algorithm gives a high Jaccard value of 0.55, a dice value of 0.70, and an F-measure value of 0.70. For this dataset, the MOPSO algorithm with three objective functions also performs well with a high F-measure value of 0.70 and a dice value of 0.70. For the macrophages segmentation dataset, the proposed algorithm gives F-measure, dice, and Jaccard values of 0.65, 0.65, and 0.48, respectively, which are the highest when compared to other algorithms. The MMPSO-S algorithm performs better than other algorithms for the neutrophils segmentation dataset. It gives F-measure, dice, and Jaccard values of 0.53, 0.53, and 0.38, respectively, which are the best values for this dataset. For the epithelial segmentation dataset, the MMPSO-S algorithm gives an F-measure, dice value, and Jaccard value of 0.63, 0.63, and 0.47, respectively, which are the highest values among all the other algorithms.

**Detection performance on the LD dataset:**

The lymphocyte detection results are given in Table 7. For this dataset, the MMPSO-S algorithm gives a precision value of 0.96, a recall value of 0.99, and an F-measure value of 0.98, which are higher than the other algorithms. The DPSO and FODPSO algorithms also exhibit a recall value of 0.99. The MOEA/D algorithm gives the lowest recall and F-measure values of 0.71 and 0.74.

**Discussion:**

The following inferences can be drawn by analysing the results from Table 5, Table 6 and Table 7. Firstly, it is observed that there is a significant difference in the Jaccard value, dice value, and F-measure value when comparing single-objective and multiobjective PSO algorithms. This shows that adding one or more suitable objective function to the PSO algorithm improves the histopathology image segmentation and detection performance.

The segmentation results given by the MOEA/D algorithm are comparatively very low for all the datasets. The F-measure and Jaccard values range from 0.03 to 0.4. The NSGA2 algorithm gives better dice, Jaccard, and F-measure values than the MOEA/D algorithm for the MONUSAC-L, MONUSAC-M, MONUSAC-N, and MONUSAC-E datasets; however, NSGA2’s performance is lower than the proposed MMPSO-S algorithm.

Secondly, it can be seen that the DPSO and FODPSO algorithms give slightly better results than the PSO algorithm for the MoNuSeg, LD, and MoNuSAC datasets. For the TNBC dataset, the PSO, the DPSO, and the FODPSO algorithms exhibit similar segmentation performance.

Thirdly, while comparing the performance of PSO variants (DPSO and FODPSO) with the multiobjective PSO algorithms, it is evident that the multiobjective algorithms provide better dice, Jaccard, and F-measure values. Among the multiobjective algorithms, MOPSO with three objective functions (Kapur + Otsu + Renyi) gives better segmentation and detection results; however, the performance of the MOPSO(Kapur + Otsu + Renyi) algorithm is slightly lower than the proposed MMPSO-S algorithm. This is because the proposed algorithm also includes the superpixel algorithm to provide better results.

Fourthly, from Table 5, Table 6 and Table 7, it is visible that the superpixel algorithm gives very low dice, Jaccard, and F-measure values as compared with single-objective PSO, DPSO, FODPSO, and multiobjective PSO algorithms. Furthermore, the superpixel technique in combination with the MMPSO-S algorithm helps in the precise refinement of ROIs and improves segmentation results. Finally, among the other multiobjective algorithms (NSGA2 and MOEA/D), the MOEA/D algorithm gives the lowest dice, Jaccard, and F-measure values for all the four datasets (TNBC, MoNuSeg, MoNuSAC, and LD). The NSGA2 algorithm gives comparatively higher values than the MOEA/D. The proposed MMPSO-S algorithm outperforms the NSGA2 and MOEA/D algorithms for all the datasets.

From Table 5, Table 6 and Table 7, it is clear that the proposed MMPSO-S algorithm yields higher segmentation and detection performance than other algorithms. The performance of the MMPSO-S algorithm is not compared with previous works on the TNBC, LD, and MoNuSAC datasets because, to the best of our knowledge, there are no recent and relevant studies using traditional image-processing methods on these datasets.

### 5.3. Normalised Execution Time

The experiments were performed on a desktop computer with an Intel(R) Xeon(R) W-2123 CPU, 16 GB of RAM, and a 1 TB hard drive, running the Windows 10 operating system. MATLAB R2019b was used to implement algorithms. The normalised execution time per 1 megapixel of the segmentation algorithms is presented in Table 8. From the table, it is clear that the superpixel algorithm has the lowest execution time compared to the other algorithms. The second best algorithm in terms of normalised execution time is the PSO algorithm with a single objective function. We know that the execution time increases as the number of objective functions increases. Hence, the proposed MMPSO-S algorithm provides a high execution time when compared with the single-objective PSO algorithm and other MOPSO variants, as it uses three objective functions along with the superpixel algorithm. On the other hand, the execution time of the MMPSO-S algorithm is lower than the DPSO and FODPSO algorithms. Considering the fact that accuracy is more important than the execution time in the histopathology image segmentation, the execution time exhibited by the proposed MMPSO-S algorithm may be acceptable.

## 6. Conclusions

This research work proposes a CAD system to detect and segment ROIs from H&E-stained histopathology images. The work demonstrates a multilevel multiobjective particle swarm optimization guided superpixel algorithm for the segmentation task. The proposed algorithm is a combination of two algorithms: (1) MMPSO algorithm with three objective functions and (2) a superpixel clustering algorithm. The MMPSO-S algorithm was tested on four different histopathology datasets. A set of experiments were conducted to evaluate the performance of the proposed algorithm in terms of segmentation results, number of thresholds, and normalised execution time. Experimental results reveal that the MMPSO-guided superpixel algorithm gives a better segmentation performance when compared to other single- and multiobjective algorithms.

## Figures and Tables

**Table 1:** The summary of the related works in image segmentation.

Algorithms	Method	Images
PSO and its variants	PSO-based Otsu’s multilevel thresholding [13]	Histopathology images
	PSO-based clustering method [14]	Histopathology images
	PSO-based multilevel thresholding [15]	Grayscale images and medical images
	Dynamic PSO [16]	Real crystal growth images
	PSO using Kapur’s and Tsallis entropy [17]	Normal brain MRI
	PSO-FCM algorithm [18]	Ultrasonic teeth images
	DPSO [19]	Satellite images
	FODPSO [20]	Infrared images
	FODPSO [21]	Retinal images
Multiobjective algorithms	UISMOPC [22]	Standard images
	MOPSO [23]	Standard images
	Multiobjective colour thresholding [24]	Standard images
	Multiobjective optimization [25]	Simulated MRI and MRI
	Multiobjective multiverse optimization [26]	Natural grayscale images
	Multiobjective grey wolf optimization [27]	Natural grayscale images
	Multiobjective artificial bee colony [28]	Standard images
Superpixel algorithm	SLIC and CNN [29]	Histopathology images
	SLIC and clustering algorithm [30]	Histopathology images
	Superpixel algorithm and clustering algorithm [31]	Satellite images
	superpixel and EM [32]	Plant disease leaves images

**Table 2:** Details of the datasets used in the work.

Dataset	Task	Total Images	Image Format
TNBC [33]	Segmentation of nuclei cells	50	.png
MoNuSeg [34,35]	Nuclei segmentation from multiple organs	44	.tif
MoNuSAC [36]	Segmentation of lymphocytes	146	.tif
	Segmentation of macrophages	58	.tif
	Segmentation of neutrophils	94	.tif
	Segmentation of epithelial cells	96	.tif
LD [37]	Detection of lymphocyte cells	100	.tif

**Table 3:** MMPSO parameter settings.

Parameters	Variable	Values
Population size	$N_{par}$	150
Maximum no. of iterations	$N_{ite}$	150
No. of thresholds	*k*	2
Position range	$\left[X_{min},X_{max}\right]$	[0, 255]
Velocity range	$\left[V_{min},V_{max}\right]$	[−5, 5]
Repository size	$A_{max}$	30

**Table 4:** Segmentation results of MoNuSeg dataset.

Algorithm	F-Measure	Dice Value	Jaccard Value
PSO	0.59	0.59	0.44
DPSO	0.62	0.62	0.45
FODPSO	0.62	0.62	0.46
MOPSO(Kapur + Otsu)	0.69	0.69	0.54
MOPSO(Renyi + Otsu)	0.61	0.61	0.45
MOPSO(Kapur + Renyi)	0.69	0.69	0.54
MOPSO(Kapur + Otsu + Renyi)	0.71	0.71	0.55
Superpixel algorithm	0.59	0.59	0.43
MOEA/D	0.38	0.38	0.24
NSGA2	0.58	0.58	0.43
Otsu threshold [52]	0.03	-	0.05
Watershed transform [52]	0.09	-	0.08
The ImageJ2-Fiji package [52]	0.18	-	0.34
Region growing [53]	-	0.37	0.16
Active contour [53]	-	0.58	0.28
**MMPSO-S**	**0.72**	**0.72**	**0.56**

**Table 5:** Segmentation results of TNBC dataset.

Algorithm	F-Measure	Dice Value	Jaccard Value
PSO	0.61	0.61	0.46
DPSO	0.61	0.61	0.46
FODPSO	0.61	0.61	0.46
MOPSO(Kapur + Otsu)	0.57	0.57	0.42
MOPSO(Renyi + Otsu)	0.63	0.63	0.46
MOPSO(Kapur + Renyi)	0.60	0.60	0.44
MOPSO(Kapur + Otsu + Renyi)	0.64	0.64	0.47
Superpixel algorithm	0.54	0.54	0.38
MOEA/D	0.24	0.24	0.14
NSGA2	0.58	0.58	0.42
**MMPSO-S**	**0.65**	**0.65**	**0.49**

**Table 6:** Segmentation results of MoNuSAC dataset.

Dataset	Algorithm	F-Measure	Dice Value	Jaccard Value
MoNuSAC-L	PSO	0.68	0.68	0.53
	DPSO	0.66	0.66	0.50
	FODPSO	0.67	0.67	0.52
	MOPSO(Kapur + Otsu)	0.68	0.68	0.52
	MOPSO(Renyi + Otsu)	0.67	0.67	0.51
	MOPSO(Renyi + Kapur)	0.67	0.67	0.51
	MOPSO(Kapur + Renyi + Otsu)	**0.70**	**0.70**	0.54
	Superpixel algorithm	0.47	0.47	0.32
	MOEA/D	0.42	0.42	0.27
	NSGA2	0.40	0.40	0.31
	**MMPSO-S**	**0.70**	**0.70**	**0.55**
MoNuSAC-M	PSO	0.55	0.55	0.38
	DPSO	0.56	0.56	0.40
	FODPSO	0.58	0.58	0.41
	MOPSO(Kapur + Otsu)	0.59	0.59	0.43
	MOPSO(Renyi + Otsu)	0.57	0.57	0.40
	MOPSO(Renyi + Kapur)	0.57	0.57	0.41
	MOPSO(Kapur + Renyi + Otsu)	0.63	0.63	0.47
	Superpixel algorithm	0.34	0.34	0.23
	MOEA/D	0.31	0.31	0.20
	NSGA2	0.62	0.62	0.48
	**MMPSO-S**	**0.65**	**0.65**	**0.48**
MoNuSAC-N	PSO	0.44	0.44	0.29
	DPSO	0.45	0.45	0.30
	FODPSO	0.47	0.47	0.31
	MOPSO(Kapur + Otsu)	0.50	0.50	0.35
	MOPSO(Renyi + Otsu)	0.51	0.51	0.36
	MOPSO(Renyi + Kapur)	0.46	0.46	0.31
	MOPSO(Kapur + Renyi + Otsu)	0.50	0.50	0.34
	Superpixel algorithm	0.44	0.44	0.29
	MOEA/D	0.12	0.12	0.07
	NSGA2	0.35	0.35	0.23
	**MMPSO-S**	**0.53**	**0.53**	**0.38**
MoNuSAC-E	PSO	0.52	0.52	0.35
	DPSO	0.57	0.57	0.40
	FODPSO	0.59	0.59	0.42
	MOPSO(Kapur + Otsu)	0.61	0.61	0.44
	MOPSO(Renyi + Otsu)	0.58	0.58	0.41
	MOPSO(Renyi + Kapur)	0.59	0.59	0.42
	MOPSO(Kapur + Renyi + Otsu)	0.62	0.62	0.46
	Superpixel algorithm	0.37	0.37	0.25
	MOEA/D	0.18	0.18	0.11
	NSGA2	0.50	0.50	0.34
	**MMPSO-S**	**0.63**	**0.63**	**0.47**

**Table 7:** Detection results of LD dataset.

Algorithm	Precision	Recall	F-Measure
PSO	0.84	0.96	0.90
DPSO	0.85	**0.99**	0.92
FODPSO	0.86	**0.99**	0.92
MOPSO(Kapur + Otsu)	0.87	0.96	0.91
MOPSO(Kapur + Renyi)	0.88	0.94	0.91
MOPSO(Renyi + Otsu)	0.87	0.96	0.91
MOPSO(Kapur + Otsu + Renyi)	0.93	**0.99**	0.96
Superpixel algorithm	0.78	0.83	0.80
NSGA2	0.86	0.94	0.90
MOEA/D	0.78	0.71	0.74
**MMPSO-S**	**0.96**	**0.99**	**0.98**

**Table 8:** Comparison of algorithms in terms of normalised execution time (in seconds) per megapixel.

Algorithm	TNBC	MoNuSeg	MoNuSAC- L	MoNuSAC- M	MoNuSAC- N	MoNuSAC- E	LD
PSO	0.021	0.036	0.022	0.021	0.021	0.038	0.011
DPSO	0.186	0.203	0.203	0.196	0.192	0.200	0.207
FODPSO	0.241	0.238	0.276	0.246	0.265	0.255	0.280
MOPSO(Kapur + Otsu)	0.102	0.098	0.104	0.102	0.092	0.091	0.098
MOPSO(Renyi + Otsu)	0.100	0.094	0.098	0.096	0.098	0.085	0.092
MOPSO(Kapur + Renyi)	0.096	0.072	0.093	0.089	0.091	0.087	0.086
MOPSO(Kapur + Otsu + Renyi)	0.112	0.103	0.084	0.110	0.095	0.097	0.101
Superpixel algorithm	0.015	0.031	0.020	0.014	0.018	0.031	0.016
MMPSO-S	0.126	0.125	0.100	0.126	0.128	0.116	0.109

## Data Availability

Not applicable.

## Appendix A. Evaluation Metrics

This section details the evaluation metrics used for evaluating the performance of the proposed method for the detection and segmentation of ROIs from histopathology images. The evaluation metrics used to measure the segmentation performance in this work are F-measure, dice coefficient, and Jaccard coefficient, which are calculated using true positive (TP), true negative (TN), false positive (FP), and false negative (FN) values. TP refers to the number of ROI pixels that are correctly predicted as ROI pixels. TN refers to the number of pixels that are correctly predicted as pixels not belonging to ROI. FP refers to the number of non ROI pixels that are incorrectly predicted as ROI pixels. FN refers to the number of ROI pixels that are incorrectly predicted as non ROI pixels.

The percentage of ROI pixels accurately identified is called sensitivity/recall and is given by Equation (A1). Precision is the ratio between ROI pixels accurately identified and the total number of pixels predicted as ROI pixels. Precision is given by Equation (A2). F-measure is defined as the harmonic mean of precision and recall measures. F-measure is given by Equation (A3).

(A1) $$Recall=\frac{TP}{TP+FN}$$

(A2) $$Precision=\frac{TP}{TP+FP}$$

(A3) $$F-measure=2\times \frac{Precision\times Recall}{Precision+Recall}$$

Jaccard coefficient compares segmented images with ground truth images by calculating the similarity between them. Jaccard value ranges between 0 and 1. A high Jaccard value indicates that the segmented result is similar to the mask. It is also known as intersection over union measure. Let *A* be the ground truth mask image, *B* be the segmented result obtained from the algorithm, |A∩B| denote the number of pixels that are common in *A* and *B*, and |A∪B| denote the number of pixels in *A* and *B*. Then, the Jaccard coefficient of *A* and *B* is given by Equation (A4).

(A4) $$\mathrm{Jaccard}\;\mathrm{Coefficient}=\frac{\mathrm{Area}\;\mathrm{of}\;\mathrm{intersection}}{\mathrm{Area}\;\mathrm{of}\;\mathrm{union}}=\frac{\left|A\cap B\right|}{\left|A\cup B\right|}$$

Dice similarity coefficient is widely used for measuring the performance of segmentation algorithms. The value of the dice similarity coefficient ranges from 0 to 1. A Dice similarity value of 1 implies a perfect segmentation while a Dice similarity value of 0 implies that there is no overlap. Let |A| be the total number of pixels in segmented image *A* and |B| be the total number of pixels in ground truth image *B*. Dice similarity coefficient for *A* and *B* is given by Equation (A5).

(A5) $$\mathrm{Dice}\;\mathrm{Coefficient}=\frac{2\times |A\cap B|}{\left|A|+|B\right|}$$

The evaluation metrics used to measure the detection performance in this work are recall, precision, and F-measure, which are calculated using true positive ($TP_{D}$), false positive ($FP_{D}$), and false negative ($FN_{D}$) values. $TP_{D}$ refers to the number of correctly detected lymphocytes. $FP_{D}$ refers to the number of incorrectly detected lymphocytes. $FN_{D}$ refers to the number of lymphocytes that are not detected. High precision and high recall imply that most lymphocytes are detected correctly. Recall, precision, and F-measure with respect to the detection are given by Equations (A6)–(A8), respectively.

(A6) $$Recall_{D}=\frac{TP_{D}}{TP_{D}+FN_{D}}$$

(A7) $$Precision_{D}=\frac{TP_{D}}{TP_{D}+FP_{D}}$$

(A8) $$F-measure_{D}=2\times \frac{Precision_{D}\times Recall_{D}}{Precision_{D}+Recall_{D}}$$
